# Negative life events and migraine: a cross-sectional analysis of the Brazilian Longitudinal Study of Adult Health (ELSA-Brasil) baseline data

**DOI:** 10.1186/1471-2458-14-678

**Published:** 2014-07-03

**Authors:** Itamar S Santos, André R Brunoni, Alessandra C Goulart, Rosane H Griep, Paulo A Lotufo, Isabela M Benseñor

**Affiliations:** 1 Departamento de Clínica Médica, Faculdade de Medicina da Universidade de São Paulo, Av. Dr. Enéas Carvalho de Aguiar, 155, 8o. andar, Bloco 3, Cerqueira César ZIP code 05403-000, São Paulo, Brazil; 2 Centre for Clinical and Epidemiological Research, Hospital Universitário da Universidade de São Paulo, Avenida Professor Lineu Prestes, 2565, 3o andar, Cidade Universitária, ZIP code 05508-000, São Paulo, Brazil; 3 Instituto Oswaldo Cruz, Fundação Oswaldo Cruz, Avenida Brasil, 4365 Manguinhos, Rio de Janeiro ZIP code 21040-360, Brazil

**Keywords:** Migraine, Life events, Epidemiology, Cross-sectional

## Abstract

**Background:**

Stress is a typical migraine trigger. However, the impact of negative life events on migraine activity is poorly studied. The aim of this study is to investigate the association between negative life events and migraine using data from the Brazilian Longitudinal Study of Adult Health (ELSA-Brasil) baseline assessment.

**Methods:**

ELSA-Brasil is a multicenter cohort study conducted in six Brazilian cities. Baseline assessment included validated questionnaires for headache classification and the occurrence of five pre-specified negative life events (financial hardship, hospitalization other than for childbirth, death of a close relative, robbery and end of a love relationship), focusing on a 12-month period before evaluation. We built crude and adjusted logistic regression models to study the association between the occurrences of negative life events and migraine diagnosis and activity.

**Results:**

We included 4,409 individuals with migraine and 4,457 participants without headache (reference). After adjustment for age, sex, race, income and educational level, we found that the occurrence of a negative life event (Odds ratio = 1.31; 95% confidence interval = 1.19 – 1.45) was associated with migraine. However, after stratifying with subgroup analyses, only financial hardship (Odds ratio = 1.65; 95% confidence interval = 1.47 – 1.87) and hospitalization (Odds ratio = 1.47; 95% confidence interval = 1.25 – 1.72) were independently associated with migraine. Further adjustment for a current major depression episode and report of religious activity did not significantly change the results. Considering migraine frequency as (a) less than once per month, (b) once per month to once per week, or (c) more than once per week, financial hardship and hospitalization remained significantly associated with migraine in all episode frequency strata, with higher odds ratios for higher frequencies in adjusted models. We also observed a significant association between the death of a close relative and the highest migraine frequency stratum (Odds ratio = 1.38; 95% confidence interval = 1.09 – 1.75) in full-adjusted model.

**Conclusions:**

The occurrence of financial hardship and hospitalization had a direct and independent association with migraine diagnosis and frequency. The death of a close relative was also independently associated with the highest migraine frequency stratum.

## Background

Negative life events (NLE), such as personal illness, death of a close relative, major financial crisis, loss/robbery of valuable things and termination of a steady relationship [1] are sources of stress in daily life. There are substantial data about the influence of the occurrence of such events on further development and recurrence of mental health disorders. Aside from the onset of post-traumatic stress disorders, NLEs may influence the course of depression [2,3], anxiety [4,5], suicidal behavior [6] and cognitive performance [7].

Patients describe stress as a common trigger for migraine episodes [8]. In a study of 1,750 migraine patients in the United States [9], 75.9% reported triggering factors, and within this group, 79.7% reported that stress was one frequent trigger. In addition, in an analysis of two-month data of headache activity, perceived stress, cognitive appraisal, and coping strategy on 20 patients who experience migraines, there were significant temporal correlations between migraine activity and daily stress [10]. In addition, fluctuations in stress levels have been proposed by others as a migraine episode trigger [11-13]. Recently, Lipton et al. [14] also described, analyzing daily data from a sample of 22 migraineurs, that higher migraine activity may actually be linked to a fluctuation in stress levels. Those authors found increased odds ratios for migraine attacks 6 to 18 hours following a decrease in stress levels.

Nonetheless, the impact of NLEs on migraine activity is less studied. A study by Reynolds and Hovanitz [15] found a mild correlation between the occurrence of NLEs and the frequency of headaches, which was no longer significant after adjustment for depression diagnosis. There was no separate analysis focusing on the migraine subgroup in that study. Hedborg et al. [16] studied the frequency of NLEs in 150 migraineurs with two or more migraine episodes per month (106 women and 44 men). In that study, a significantly higher proportion of women reported such events during adulthood, compared with men (79.2% vs. 52.3%, p = 0.001). These differences were mainly due to the report of NLEs involving a close relative (diseases, accidents or death), work (conflicts, lack of control or new job) and bullying. Scher et al. [17] studied 206 individuals with chronic daily headache (CDH) and 507 episodic headache controls, all participants from the Frequent Headache Epidemiology Study. They found a positive association between chronic daily headache and the occurrence of major life events (work changes, relationship changes, major changes with children, changes in residence, deaths of family members or close friends and self-defined extremely stressful situations) in the same year or the year before CDH onset compared to those with episodic headache (Odds Ratio (OR) = 1.20; p < 0.001). However, when the analyses were limited to those with migraine, this association had borderline significance (OR = 1.15; p = 0.059). A prospective study by Larsson et al. [18] showed that reduced leisure time activities were associated with higher migraine frequency, in models including the presence of depressive symptoms.

The Brazilian Longitudinal Study of Adult Health (ELSA-Brasil) is a multicenter study that includes civil servants from six cities in three regions in Brazil (South, Southeast, and Northeast). Brazil is a country with a high prevalence of migraine [19,20], and this setting was rarely studied in previous works assessing the association between negative life events and migraine. In addition, ELSA-Brasil used a structured, validated questionnaire for migraine diagnosis according to the IHS criteria, avoiding bias associated with self-reporting of medical diagnosis [21]. The aim of the present study is to evaluate the association, during the year before baseline assessment, between the occurrence of NLEs and migraine in the ELSA-Brasil cohort participants.

## Methods

### Study design

The ELSA-Brasil design and concepts have been detailed elsewhere [22]. Briefly, it is a cohort study of 15,105 civil servants from six cities (São Paulo, Belo Horizonte, Porto Alegre, Salvador, Rio de Janeiro, and Vitoria) that is focused primarily on cardiovascular diseases and diabetes. The baseline assessment took place from August 2008 to December 2010. All active or retired employees of the six cities, aged between 35 and 74 years, were eligible for the study. In baseline assessment, trained personnel conducted in-person interviews, including a validated questionnaire for migraine diagnosis and data on five pre-specified NLEs (financial hardship, hospitalization other than for childbirth, death of a close relative, robbery and end of a love relationship), described in detail below. Clinical and laboratory measurements were also performed [23].

From 15,105 ELSA-Brasil participants, we excluded from analyses 12 (0.08%) who did not answer the headache questionnaire, 6,222 (41.2%) who had headaches not classifiable as migraine and 5 (0.03%) who did not have information on the occurrence of life events. Therefore, our sample for this study included 4,457 individuals without headache and 4,409 with migraine.

### Headache questionnaire and migraine diagnosis

All of the participants who answered “yes” to the question, “In the last 12 months, did you have a headache?” at the ELSA-Brasil baseline evaluation were invited to answer a detailed headache questionnaire based on the second version of the International Headache Society criteria [24]. This questionnaire is validated in Brazilian Portuguese [25] and has been used in previous studies [26,27]. Briefly, it assesses pain frequency, duration, quality, location, intensity, triggering factors, and accompanying symptoms, such as nausea or vomiting and the presence of aura. Migraine was defined as the presence of definite migraine (IHS reference codes 1.1-migraine without aura or 1.2-migraine with aura) or probable migraine (IHS reference code 1.6).

### Life events questionnaire

The ELSA-Brasil baseline assessment included questions based on a validated questionnaire [28] addressing the occurrence of negative, stressful life events. The following five questions were used: (a) “In the last 12 months, did you face a financial hardship more serious than usual?”; (b) “In the last 12 months, were you hospitalized for one night or more, due to illness or accident (except childbirth)?”; (c) “In the last 12 months, did a close relative (parent, spouse, partner, child or sibling) die?”; (d) “In the past 12 months, were you robbed, that is, had money or any goods taken by the use or threat of violence, or physical aggression?” and (e) “In the last 12 months, did you suffer any disruption of a loving relationship, including divorce or separation?” Whenever participants responded positively to any of these questions, additional information about frequency was gathered.

### Other variables

Age was stratified in 10-year intervals (35–44, 45–54, 55–64, 65–74). Information on educational level and net family income was provided by participants according to pre-defined stratification. Local currency (Brazilian Reais, BRL) was converted to US dollars (USD) at a rate of BRL 2.00 = USD 1.00. We assessed religious activity during the past 12 months by analyzing participants’ answers to the question: “In the last 12 months (excluding situations such as a wedding, christening or funeral), did you attend religious services, church services or activities of your religion or another religion?” Race was self-defined according to the following question: “*The Brazilian Census uses some terms, Black, Mixed (pardo), White, Asian, and Native, to classify an individual’s race. If you had to respond to the Brazilian Census today, how would you classify your race?*” For this study, Asians and individuals of Native ancestry were grouped as “others” because the number of individuals in these categories was small.

Mental diagnoses were assessed by trained interviewers using an adapted Brazilian-Portuguese version of the Clinical Interview Schedule – Revised (CIS-R). The CIS-R is a structured interview for measurement and diagnosis of community non-psychotic psychiatric morbidity. It was developed specifically to be used in community and primary care, being a short and straightforward questionnaire [29]. Importantly, lay interviewers are as reliable as psychiatrists in using CIS-R for performing mental diagnosis, making it a suitable instrument to be used in our cohort. The CIS-R yields diagnoses according to the tenth revision of the International Classification of Diseases. In this study, we used the category “depression” (all types and severities) to explore its relationship with our hypothesis.

### Statistical analysis

We describe the groups of individuals without headache and with migraine according to age strata, sex, race, educational level, net family income, the occurrence of NLEs, religious activity and major depression diagnosis. We performed chi-square tests to study the association between the occurrence of life events (in separate and all together) and migraine in the year before baseline examination. We created binary logistic regression models to determine whether the occurrence of financial hardship, hospitalization, death of a close relative, robbery, end of a love relationship or any of these events during the year before assessment was related to migraine and its activity. First, we considered migraine diagnosis, regardless of frequency, as the dependent variable. Models are presented (1) as a crude rate, (2) adjusted for age, sex, race [30], income [31] and educational level [32] and (3) full-adjusted for age, sex, race, income, educational level, major depressive episode diagnosis and the report of current religious activity. We included these two last variables to identify whether they influence the association between NLEs and migraine, based on interaction and non-interaction models. We also built models considering migraine frequency as the dependent variable: (1) less than one episode per month, (2) from one episode per month to one episode per week and (3) more than one episode per week. For the analyses using migraine frequency information, we present crude and full-adjusted models. For all models, we considered individuals without headache as the reference group. We also ran sensitivity analyses restricting migraine cases to IHS reference codes 1.1 and 1.2 (definite migraine). The significance level was set at 0.05. We performed analyses using R for Windows version ×64 2.15.1 [33] with the epicalc [34] package.

### Ethical considerations

The study protocol conforms to the Declaration of Helsinki. The Institutional Review Boards (IRB) that approved the study protocol in each of the six study centers were: Hospital Universitário da Universidade de São Paulo IRB (Universidade de São Paulo), Fundação Oswaldo Cruz IRB (Fundação Oswaldo Cruz), Instituto de Saúde Coletiva da Universidade Federal da Bahia IRB (Universidade Federal da Bahia), Universidade Federal de Minas Gerais IRB (Universidade Federal de Minas Gerais), Centro de Ciências da Saúde da Universidade Federal do Espírito Santo IRB (Universidade Federal do Espírito Santo) and Hospital de Clínicas de Porto Alegre IRB (Universidade Federal do Rio Grande do Sul). All individuals who participated in the study provided written informed consent.

## Results

Table 1 shows the baseline characterization of both groups (no headache and migraine) in bivariate analyses. Participants with migraine were younger, more prone to be of female sex and of lower net family income strata. In addition, a higher proportion of migraineurs stated that there had been a NLE in the year before the baseline assessment. This difference was mainly due to the report of the specific life events financial hardship (P < 0.001), hospitalizations other than for childbirth (P < 0.001) and the end of a love relationship (p = 0.003).

**Table 1:** Baseline characteristics of study participants according to migraine status

	**No headache N = 4,457**	**Participants with migraine**		
		**Definite N = 1,265**	**Probable N = 3,144**	**All N = 4,409**
Age strata				
35-44	568 (12.7)	363 (28.7)	837 (26.6)	1,200 (27.2)
45-54	1,430 (32.1)	617 (48.8)	1,388 (44.1)	2,005 (45.5)
55-64	1,593 (35.7)	241 (19.1)	769 (24.5)	1,010 (22.9)
65-74	866 (19.4)	44 (3.5)	150 (4.8)	194 (4.4)
Female sex	1,698 (38.1)	1,105 (87.4)	2,256 (71.8)	3,361 (76.2)
Race				
White	2,186 (49.9)	618 (49.2)	1,596 (51.3)	2,214 (50.7)
Mixed (Pardo)	1,266 (28.9)	352 (28.0)	907 (29.2)	1,259 (28.8)
Black	753 (17.2)	244 (19.4)	506 (16.3)	750 (17.2)
Other	178 (4.1)	42 (3.3)	102 (3.3)	144 (3.3)
Educational level				
Lower than High School	772 (17.3)	122 (9.6)	352 (11.2)	474 (10.8)
High school	1,496 (33.6)	537 (42.5)	1,207 (38.4)	1,744 (39.6)
College or above	2,189 (49.1)	606 (47.9)	1,585 (50.4)	2,191 (49.7)
Net family income				
< USD 1245	1,263 (28.5)	399 (31.6)	874 (27.9)	1,273 (29.0)
USD 1245 – 3319	1,824 (41.1)	591 (46.8)	1,488 (47.5)	2,079 (47.3)
> = USD 3320	1,348 (30.4)	272 (21.6)	770 (24.6)	1,042 (23.7)
Stressful life events				
Any event	1,754 (39.4)	638 (50.4)	1,492 (47.5)	2,130 (48.3)
Financial hardship	738 (16.6)	382 (30.2)	827 (26.3)	1,209 (27.4)
Hospitalization	418 (9.4)	167 (13.2)	383 (12.2)	550 (12.5)
Death of a close relative	521 (11.7)	139 (11.0)	342 (10.9)	481 (10.9)
Robbery	283 (6.3)	86 (6.8)	228 (7.3)	314 (7.1)
End of a love relationship	248 (5.6)	88 (7.0)	227 (7.2)	315 (7.1)
Religious activity in the past year	2,951 (66.2)	999 (79.0)	2,301 (73.2)	3,300 (74.9)
Major depressive disorder	101 (2.3)	129 (10.2)	242 (7.7)	371 (8.4)
Migraine frequency				
Less than once per month		291 (23.1)	1,384 (44.1)	1,675 (36.1)
From once per month to once per week		573 (45.4)	1,140 (36.3)	1,713 (38.9)
More than once per week		397 (31.5)	615 (19.6)	1,012 (23.0)

Sum may exceed 100% because of rounding. Definite migraine was defined as IHS reference codes 1.1 or 1.2. Probable migraine was defined as IHS reference code 1.6 [24].

The results of the logistic regression models, considering migraine diagnosis as the dependent variable, are shown in Table 2. Migraine was significantly associated with the occurrence of financial hardship and hospitalization in the year before assessment. These associations remained significant in full-adjusted models. Considering migraine diagnosis regardless of frequency, the death of a close relative, robbery and the end of a love relationship had no independent associations with the occurrence of migraine. In addition, full-adjusted models built considering interactions between major depressive disorder or religious activity and NLEs did not reveal any significant interaction terms. When sensitivity analyses restricted migraine cases to those with definite migraine (IHS codes 1.1 or 1.2), these results did not significantly change.

**Table 2:** Odds ratios (95% confidence intervals) for the association between NLEs and migraine episodes

	**Crude model**	**Adjusted for age, sex, race, income and educational level**	**Full-adjusted model**^**a**^
Any event	1.44 (1.32 – 1.57)***	1.31 (1.19 – 1.45)***	1.26 (1.14 – 1.39)***
Financial hardship	1.90 (1.72 – 2.11)***	1.65 (1.47 – 1.87)***	1.56 (1.38 – 1.76)***
Hospitalization	1.38 (1.20 – 1.58)***	1.47 (1.25 – 1.72)***	1.41 (1.20 – 1.65)***
Death of a close relative	0.93 (0.81 – 1.06)	1.07 (0.92 – 1.24)	1.06 (0.91 – 1.24)
Robbery	1.13 (0.96 – 1.33)	1.02 (0.84 – 1.24)	0.99 (0.82 – 1.21)
End of a love relationship	1.31 (1.10 – 1.55)**	0.93 (0.76 – 1.14)	0.87 (0.71 – 1.07)

^a^Adjusted for age, sex, race, income, educational level, major depressive episode diagnosis and the report of current religious activity. **p < 0.01. ***p < 0.001.

Table 3 shows the results of logistic regression models in which we stratified participants with migraine according to episode frequency. Financial hardship and hospitalization remained significantly associated with migraine in all episode frequency strata. We observed higher point OR estimates for both financial hardship and hospitalization as we targeted analyses toward individuals with higher migraine episode frequencies. Also, we verified a positive association between the highest migraine frequency stratum and death of a close relative during the past year.

**Table 3:** Odds ratios (95% confidence intervals) for the association between NLEs and each migraine frequency stratum

	**Crude models**			**Full-adjusted models**^**a**^		
**Migraine frequency**	**< Once per month**	**From once per month to once per week**	**> Once per week**	**< Once per month**	**From once per month to once per week**	**> Once per week**
Any event	1.27 (1.13 – 1.42)***	1.32 (1.18 – 1.48)***	2.06 (1.79 – 2.37)***	1.15 (1.02 – 1.31)*	1.17 (1.02 – 1.34)*	1.61 (1.37 – 1.90)***
Financial hardship	1.59 (1.38 – 1.82)***	1.79 (1.57 – 2.05)***	2.76 (2.37 – 3.21)***	1.33 (1.14 – 1.55)***	1.48 (1.26 – 1.75)***	1.93 (1.60 – 2.32)***
Hospitalization	1.30 (1.09 – 1.56)**	1.22 (1.02 – 1.46)*	1.80 (1.48 – 2.19)***	1.28 (1.05 – 1.56)*	1.28 (1.03 – 1.59)*	1.74 (1.36 – 2.22)***
Death of a close relative	0.88 (0.74 – 1.06)	0.84 (0.70 – 1.01)	1.15 (0.94 – 1.41)	0.96 (0.79 – 1.17)	1.08 (0.87 – 1.33)	1.38 (1.09 – 1.75)**
Robbery	1.16 (0.93 – 1.44)	0.98 (0.78 – 1.24)	1.35 (1.05 – 1.74)*	1.08 (0.85 – 1.38)	0.82 (0.63 – 1.08)	1.10 (0.81 – 1.50)
End of a love relationship	1.17 (0.93 – 1.48)	1.29 (1.03 – 1.62)*	1.56 (1.20 – 2.01)***	0.91 (0.70 – 1.18)	0.79 (0.60 – 1.03)	1.00 (0.73 – 1.36)

^a^Adjusted for age, sex, race, income, educational level, major depressive episode diagnosis and the report of current religious activity. *p < 0.05. **p < 0.01. ***p < 0.001.

Restricting migraine cases to those with definite migraine and considering each frequency stratum in full-adjusted models, we found a positive association between financial hardship and definite migraine in the two highest frequency strata (OR = 1.51; 95% CI = 1.18 – 1.93 for migraine episodes from once per month to once per week and OR = 1.67; 95% CI = 1.28 – 2.18 for migraine episodes more than once per week). For the association between hospitalization and definite migraine, we found a significant positive association in sensitivity analysis adjusted models only for the highest frequency stratum (OR = 1.54; 95% CI = 1.09 – 2.19). For the association between death of a close relative and definite migraine with episodes occurring more than once a week, statistical significance remained in sensitivity analysis (OR = 1.49; 95% CI = 1.05 – 2.11). However, for all cases in which a loss of statistical significance occurred, point OR estimates for the association with all migraine and definite migraine were very similar, and the loss of significance may be related to the smaller number of definite migraine cases.

## Discussion

We found significant, direct associations between migraine headache, as well as its frequency and financial hardship and hospitalizations (for reasons other than childbirth) in the year before the ELSA-Brasil baseline assessment after multivariate adjustment. Also, analyses of individuals in the highest migraine frequency stratum (more than once per week) showed a significant association between migraine and the death of a close relative in the past year. In addition, further adjustment for the report of religious activity and major depression did not significantly change the results.

Although the link between stressors and negative health outcomes is not completely understood, some authors have proposed mechanisms to explain this association. One possible explanation is that the influence of such events on physical health is mediated by the development of depressive and other psychiatric symptoms [3,35]. However, the presence of major depressive disorder did not influence this association, suggesting that other mechanisms of association may be present. For instance, other authors have highlighted biological pathways to explain the connection between NLEs and physical health. Wittstein et al. [36] reported a series of cases of cardiac dysfunction precipitated by acute emotional stress, known as the “broken heart” syndrome. Those authors suggested that this syndrome could be related to acute sympathetic stimuli, a neuromodulation pathway also potentially involved in migraine pathophysiology [37]. Similarly, the activation of the hypothalamus-pituitary-adrenal axis in stressful situations [38,39] may also be associated with a higher migraine activity [40]. In addition, there is some evidence that migraineurs may have stronger and more sustained responses after emotional distress [41,42], which may predispose these individuals to more deleterious effects caused by stressful situations. This is compatible to the recent concept of allostatic load, in which body responses targeted to compensate and adapt to new situations (as the occurrence of stressors) may accumulate overtime and eventually result in a maladaptive new steady-state [43]. On the other hand, a recent study by Lipton et al. [14] suggests that fluctuations in stress levels, rather than the absolute perceived stress levels (measured by daily report of the Perceived Stress Scale and the Self-Reported Stress Scale scores) may be an episode trigger. Unfortunately, we cannot explore further this temporality with our data, as it would require a daily record of migraine activity and perceived stress.

In our study, some life events were associated with migraine, whereas others were not. Financial hardship was the NLE with the strongest association with migraine activity in our study, even in full-adjusted models that included family income and depression diagnosis. Lynch et al. [44] also studied the association between financial hardship and health. Analyzing income data from 1,124 individuals between 1965 and 1983 and physical and psychological functional outcomes 11 years later, they found an increased frequency of depressive symptoms and reduced performance in daily living activities in those who reported more often financial difficulties during that period.

In addition to an association mediated by a higher level of stress caused by financial hardship, and thus higher migraine activity, financial hardship driven by loss of productivity, absenteeism and money spent on medicine because of a higher level of migraine activity may also be mechanisms underlying the association. However, characteristics of our cohort make this reverse causation less probable. The ELSA-Brasil is a multicenter cohort of formally employed civil servants, who are protected by Brazilian laws of the labor force. This is similar to a tenure track, which prevents against income loss because of health-related absenteeism. Most of them are not subject to productivity rules and earn a fixed monthly salary. Yet, some, although not all, medications used for migraine treatment, including prophylaxis, are available free of charge in the country, upon medical prescription. Finding a positive association in this setting is a strong argument that the predominant mechanism of association between financial hardship and migraine are not because of reverse causation and, rather, may be related to higher stress levels.

Hospitalizations for reasons other than childbirth were also associated with migraine in our study, and this association was stronger with higher migraine activity. Although some may advocate that headache attacks could motivate a higher number of hospitalizations, this explanation seems unlikely. Hospitalization because of migraine is not very common. Hawkins et al., studying paid claims for migraine treatment for a total of 215,209 US employees with migraine diagnosis in 2004, found that only a very small amount of the direct related health costs for this condition were relate to inpatient care [45].

We also found a significant, positive association between death of a close relative and the highest migraine frequency stratum. Also, increasing migraine frequency led to increasing point OR estimates for this association, although this was non-significant in the first two migraine frequency groups. Although our data do not allow to make undoubted affirmatives, it seems reasonable that some positive association exists between those conditions. During grief, the occurrence of somatic symptoms, including headaches, has been recognized for decades [46,47]. Surprisingly, with the exception of small studies [48], the association between the loss of a family member and migraine activity is poorly studied.

Robbery and the end of a love relationship had no clear association with migraine activity. Circumstances that motivate financial hardship [49], hospitalizations [50] and the consequences of the death of a close relative [51] may be, in general, more perennial. Although studies about the effect of specific life events on migraine are scarce, our findings are mostly consistent with a recent study by Wardenaar et al. [52], evaluating the relationship between the occurrence of life events and psychiatric symptomatology. Those authors found that general distress was independently associated to the report of financial problems and illness/injury/victimization. Robbery and the death of a close relative were not associated to general distress in their study. In addition, as pointed out earlier, more recent work on allostatic load includes a new variable to this model, and reinforce the importance of more perennial causes of stress. We may speculate that stress-mediated changes caused by such events may induce a more sustained body response and be related to higher migraine activity. These body changes, according to the allostatic load theory [43], may trigger a maladaptive cycle and persist even after the cessation of the stressor.

There is another possible explanation for the differences observed for the associations among the studied NLEs and migraine. The influence of causes of persistent stress are more likely to be identified in a cross-sectional study evaluating migraine activity during a 12-month period. We cannot exclude the hypothesis that robbery and the end of a love relationship may have led to a transient increase in migraine activity that was not sufficient to alter the participants’ perception for the whole 12-month period.

We found, for financial hardship, hospitalization other than for childbirth and death of a close relative, increasing odds ratios associated with higher migraine activity. For all of them, the highest migraine frequency stratum (> once per week) was associated with the highest odds ratios. This is consistent to the findings from two other studies. Scher et al. [17] analyzed individuals with CDH and episodic headache, and found that the occurrence of major life events were associated with headache chronicity. However, in that study, when the analyses were restricted to those with migraine, there was a trend towards a positive association, but with borderline significance (OR 1.15; p = 0.059). We may speculate, from the results of their study and ours, that the occurrence of negative life events may increase migraine activity. Larsson et al. [18] analyzed predictors of frequent headache (≥ 1 episode per week) in 2,355 teenagers during a one-year follow-up. Those authors found that, in a model including the reduction of leisure time activities, levels of depressive symptoms and gender as independent variables, all of them were significant predictors of frequent headache at follow-up.

The effects of religious attendance on counteracting putative deleterious effects on health outcomes mediated by the occurrence of NLEs have been studied by others, with conflicting results. Bradshaw and Ellison [53] found religious attendance to attenuate the effects of objective and subjective financial hardship on psychological distress. On the other hand, Kidwai et al. [54], studying the effect of religious attendance on the association between NLEs and psychological distress, did not find significant influences. In our study, we did not find such effects either, considering any specific life event nor all together. Although this is still a poorly understood field, these conflicting findings may be due to variations in study settings. For example, social support may moderate the effects of religion. There are variations on how these religion-based net supports act across different communities, and consequently, their influences on individual health are heterogeneous [55]. It is important to emphasize that it was beyond the scope of our study to investigate other social and leisure activities related to stress management. We do not draw conclusions from the present work for about the effects of stress management techniques as coping strategies for the occurrence of NLEs nor their relationship with migraine.

This study has some limitations. Because we are analyzing cross-sectional data (as we only have baseline ELSA data at the present moment), it is not possible to infer causality. However, we could adequately study the association between migraine and NLEs, and given the cohort characteristics, the finding of a positive association between migraine and financial hardship is a strong argument toward a stress-related increase in the number of migraine attacks. The questionnaires required individuals to characterize headache episodes and NLEs in the 12 months before the interview, which could have led to recall bias. Our migraine frequency question do not allow to analyze, in separate, those individuals with chronic migraine, (defined by the IHS criteria as more than 15 days/month). As stated earlier, more subtle, transient increases in migraine activity caused by experiencing robbery, death of a relative or the end of a love relationship could remain unidentified by the approach in this study.

## Conclusions

We found that financial hardship and hospitalizations (excluding for childbirth) had a positive association with migraine, after adjustment for age, sex, race, income and educational level among the ELSA-Brasil participants. Associations were stronger in groups with higher migraine episode frequency. In addition, death of a close relative in the past year was significantly and independently associated with the higher migraine frequency strata. The presence of a major depression episode or the report of religious attendance did not influence this association. These epidemiological findings in this large sample broadens the current knowledge on NLE-associated morbidity. Also, future directions of research include the determination of the pathophysiological ways that mediate the association between NLEs and migraine.

## Abbreviations

OR: Odds ratio; 95% CI: 95% confidence interval; NLE: Negative life events; CDH: Chronic daily headache; IHS: International Headache Society; US: United States; USD: American dollars; CIS-R: Clinical Interview Schedule – Revised; IRB: Institutional Review Boards.

## Competing interests

The authors declare that they have no competing interests.

## Authors’ contributions

ISS participated in study design, statistical analysis, data interpretation and wrote the paper. ARB participated in statistical analysis, data interpretation and contributed with intellectual content to the paper. ACG, RHG and PAL participated in study design and contributed with intellectual content to the paper. IMB participated in study design, data interpretation and helped to write the paper. All authors read and approved the final manuscript.

## Pre-publication history

The pre-publication history for this paper can be accessed here:

http://www.biomedcentral.com/1471-2458/14/678/prepub
